# Using geospatial modelling to estimate the prevalence of adolescent first births in Nepal

**DOI:** 10.1136/bmjgh-2018-000763

**Published:** 2019-07-01

**Authors:** Sarah Neal, Corrine Warren Ruktanonchai, Venkatraman Chandra-Mouli, Chloe Harvey, Zoe Matthews, Neena Raina, Andrew Tatem

**Affiliations:** 1 Social Statistics and Demography Department, University of Southampton, Southampton, UK; 2 WorldPop, Department of Geography and Environmental Science, University of Southampton, Southampton, UK; 3 Department of Reproductive Health and Research, World Health Organization/Human Reproduction Programme, World Health Organization, Geneva, Switzerland; 4 Social Statistics and Demography Department, University of Southampton, Southampton, UK; 5 Regional Office for South-East Asia (SEARO), World Health Organisation, New Delhi, India

**Keywords:** adolescent, sexual health, pregnancy, nepal, GIS, inequities, spatial modelling

## Abstract

**Introduction:**

Adolescent pregnancy is associated with significant risks and disadvantages for young women and girls and their children. A clear understanding of population subgroups with particularly high prevalence of first births in adolescence is vital if appropriate national responses are to be developed. This paper aims to provide detailed data on socioeconomic and geographic inequities in first births to adolescents in Nepal, including wealth quintile, education, rural/urban residence and geographic region. A key element is the use of geospatial modelling to develop estimates for the prevalence of adolescent births at the district level.

**Methods:**

The study uses data from the 2011 Nepal Demographic and Health Survey. Initial cross-tabulations present disaggregated data by socioeconomic status and basic geographic region. Estimates of prevalence of adolescent first births at the district level are creating by regression modelling using the Integrated Nested Laplace Approximation package in R software.

**Results:**

Our findings show that 40% of women had given birth before the age of 20 years, with 5% giving birth before 16 years. First births to adolescents remain common among poorer, less educated and rural women. Geographic disparities are striking, with estimates for the percentage of women giving birth before 20 years ranging from 35% to 53% by region. District level estimates showed even more marked differentials (26%–67% had given birth by 20 years), with marked heterogeneity even within regions. In some districts, estimates for the prevalence of first birth among the youngest age groups (<16 years) are high.

**Conclusion:**

Important geographic and socioeconomic inequities exist in adolescent first births. In some districts and within some subgroups, there remain high levels of adolescent first births, including births to very young adolescents. The use of Bayesian geospatial modelling techniques can be used by policymakers to target resources.

**Key messages**

**What is already known?**

- Adolescent motherhood remains a challenge in many low-income and middle-income countries and affects both the health and economic prospects of young women and their children.
- Inequity exists in many countries in the distribution of adolescent first births based on socioeconomic and geographic factors.

**What are the new findings?**

- There is marked socioeconomic and geographic variation in the distribution of the proportion of women giving birth before the age of 20 years in Nepal.
- In Nepal, estimates for adolescent first births remain very high in some districts (over 65%) and are also high among women under 16 years of age.

**What do the new findings imply?**

- Policymakers should focus efforts on reducing adolescent first births on districts where prevalence remains high, particularly for women under 16 years.
- High resolution mapping can help direct scarce resources to where they are most needed.

## Introduction

Adolescent pregnancy continues to be a threat to the health and well-being of young women and their children. In addition, education and future economic prospects of young women can be restricted by early childbearing, and this often perpetuates the cycle of poverty and deprivation,^1^ resulting in poorer health, education and life chances for the child.^2–4^ Its importance as a barrier to development is recognised through the inclusion of the adolescent fertility rate as an indicator for Goal 3 of the Sustainable Development Goals: Good Health and Wellbeing.^5^ However, reducing adolescent births has the potential for far greater impact outside the health sphere, including improving access to education, reducing poverty, reducing inequalities (including gender inequality) and promoting economic growth.^1 6^

Nepal has made some progress in reducing adolescent births; age-specific fertility rates for women aged 15–19 years have fallen from 126 per thousand in 1960 to 71 in 2015.^7^ However, progress has not been as fast or as great as in some other neighbouring countries such as India and Pakistan.^7^ Barriers to reduction include low female social status and autonomy, a culture of secrecy around sex and reproductive health and the persistence of early marriage.^8–10^ In Nepal, like much of South Asia, the majority of births occur within the context of marriage,^11 12^ so these two factors are intrinsically linked.

It is important to recognise that the national aggregate measures of adolescent childbearing such as fertility rate for women aged 15–19 years mask important socioeconomic and geographic inequities as well as important age-related data. As with other countries, the prevalence of adolescent pregnancy within particular communities or populations within Nepal are influenced by cultural norms and a range of socioeconomic factors, including education, region and place of residence, ethnicity and wealth status.^9 10^ This paper aims to provide detailed data on socioeconomic and geographic inequities in adolescent first births in Nepal, including wealth quintile, education, rural/urban residence and geographic region. A key objective of the work is to develop district level estimates of the prevalence of adolescent first births based on the 75 second level administrative boundaries to allow identification of disparities at small area level. Specifically, we use Bayesian model-based geostatistics to generate maps of adolescent first births or estimates at unsampled areas smaller than those currently provided through the Demographic and Health Surveys (DHS) programme, namely at the administrative level 3 unit.^13^

Marked spatial inequalities in prevalence of adolescent pregnancies are common in both low-income and higher-income countries and are underpinned by geographic patterns of deprivation as well as cultural norms and practices within communities. These factors can lead to hidden pockets of high levels of adolescent fertility within small geographic areas, particularly for pregnancies among very young adolescents which are rarer and tend to reflect specific local sociocultural characteristics.^14^ Data sampling, however, tends to occur at the national or regional level, necessitating the use of spatial statistics to infer estimates at a higher resolution scale. The mapping and collection of data at a more local level has been a key component of programmes to address adolescent pregnancies in high-income countries, for example and has contributed to the success of England’s Pregnancy Reduction strategy.^15^

Our study provides valuable information for policymakers in Nepal. In 2015, a review of the Adolescent Sexual and Reproductive Health Programme of Nepal suggested the need to focus on subpopulations that were failing to benefit from services due to health systems, geographical and cultural barriers.^16^ These findings contribute to a more nuanced understanding of which groups are continuing to face poor reproductive outcomes and where they are located. No prior study has examined district-level inequities for adolescent births for Nepal, so this paper will provide a significant and highly policy relevant contribution to knowledge. The ongoing decentralisation process in Nepal makes district-level data of particular importance for decision making, although the process has been limited by a number of factors.^17^

All our results are disaggregated by age at birth into three groupings: under 16 years, 16–17 years and 18–19 years. There is clear evidence that the health risks associated with adolescent motherhood is particularly concentrated among the youngest age group,^18 19^ so disaggregated adolescent first births by age enables younger adolescents to be identified and mapped separately.

## Methods

### Data

Data used for these analyses were obtained from the 2011 Nepal DHS.^20^ DHS surveys are nationally representative surveys providing estimates for a range of health and demographic indicators comparable across place and time based on a two-stage stratification process. Initial cross-tabulations of first births under the age of 20 years disaggregated by wealth quintile, urban/rural residence, marital status, highest education and region were based on a sample of women aged 20–29 years at the time of survey. DHS sampling weights were applied to these cross-tabulations to ensure the estimates were representative, thus eliminating the effect of oversampling certain populations. To do this, the ‘svyset’ function in STATA was used to adjust for the complex sampling techniques used in the DHS. Adolescent first births were disaggregated for the three topographical regions; the Terai (the lowland area in the south running the length of the country, from west to east); the Hill area (which is the central part of the country and running the full length of the country) and the Mountain region in the North. In addition, we estimated levels of first births for the five development regions as identified in the DHS: Far Western, Mid-Western, Western, Central and Eastern. Of note, DHS sampling weights were applied only to the cross-tabulation estimates in these analyses, as sampling weights are made to be representative at the DHS region or national level. For geospatial analyses, raw cluster data were used after accounting for cluster displacement, as outlined below.

All estimates were disaggregated into age groups for first birth of < 16 years, 16–17 years and 18–19 years. The use of < 16 years differs from more common usage of <15 years as the youngest group, as evidence suggests that the health risks of adolescent motherhood are raised in the<16 age group, thus making this grouping more appropriate to identify the most vulnerable.^18 21^

Data management for the district-level estimates was performed using SAS V.9.4 software, while multivariate analysis was conducted using R software.^22 23^ Women aged 20–29 years at the time of survey with corresponding geo-located DHS clusters were included in this sample, resulting in a sample size of n=4419 women. We obtained Global Positioning Systems coordinates for n=289 cluster locations through the DHS programme and mapped these locations within ArcGIS V.10.2.2 software.^24^ To protect participant confidentiality and maintain anonymity, the DHS programme randomly displaces cluster coordinates by up to 2 km for urban clusters and 5 km for rural clusters, potentially resulting in displacement bias.^25^ Therefore, according to DHS guidelines, we drew corresponding buffers around coordinate locations of 2 km and 5 km for urban and rural clusters, respectively, and geographically linked clusters to the corresponding administrative III unit or district level, with the greatest overlap for later analysis.^26^ Administrative unit shapefiles were obtained through the freely available Database of Global Administrative Units, hosted through the DIVA-GIS project (http://www.diva-gis.org), and represent the 75 Nepalese districts which existed in 2011, at the time these data were collected.^27^ As of late 2015, an additional two districts were added, resulting in 77 districts among seven provinces—this geographic change and year of DHS data collection therefore limit findings temporally to pre-2015.

### Geospatial logistic regression modelling

To estimate adolescent motherhood at the administrative III unit and assess risk factors, we employed a Bayesian geospatial logistic regression model using the Integrated Nested Laplace Approximation (INLA) package in R software.^28^ Bayesian inference has been used within the DHS literature previously in order to control for the correlation of spatial and temporal effects inherent within the nested survey design of the DHS as well as to allow for quantification of uncertainty surrounding posterior estimates.^29–33^ Briefly, a Bayesian framework generates a distribution of estimates (or posterior estimates), which can be used to present point estimates such as mean, median and mode or also SD of estimates. This allows for quantification of uncertainty, with the ‘true’ estimate falling within a range of possible values as specified through the posterior distribution. This is particularly advantageous when modelling DHS data at a different spatial scale than the spatial scale that surveys were designed to represent, such as the national and regional scale. These posterior distributions may then be visualised using Geographic Information Systems or other mapping software. This is particularly important when working with relatively rare events such as very early adolescent motherhood, which might have a wide range of surrounding uncertainty, and presents a useful way of communicating this uncertainty to policymakers. Here, we visualise uncertainty as the width of the 95% credible interval, a technique which is also regularly employed by the DHS programme when quantifying associated uncertainty in modelled surfaces.^13 25^

Within our models, the dependent variables of interest were age at first birth at: (1) less than 16 years of age, (2) between 16 and 17 years of age, (3) between 18 and 19 years of age and (4) less than 20 years of age. These groupings have been used previously within the literature and have been shown to have important differences in age patterns.^32 34^ To predict these outcomes at a spatially disaggregated scale, we employed a Besag model incorporating both spatial effects plus random effects within the INLA package, allowing for representation of the spatial component of outcome variability.^35 36^ Similar to other models in the literature,^33^ our model can therefore be defined as:

$logit\left(p_{ij}\right)=\beta_{0}+\beta_{1}x_{ij}+\beta_{2}x_{ij}\ldots +\beta_{k}x_{ij}+f_{spat}\left(admin_{j}\right)$,

where $\left(p_{ij}\right)$ represents the probability of a woman, *i,* in administrative unit, *j*, having her first child at the corresponding age group; $\beta_{0}+\beta_{1}x_{ij}+\beta_{2}x_{ij}\ldots +\beta_{k}x_{ij}$ represents a vector of the fixed effects of predictors on the dependent variable of interest and $f_{spat}\left({admin}_{j}\right)$ represents the spatial effect of the administrative unit, *j*, and random effects and can therefore be further subdivided into both structured and random unstructured effects:

$$f_{spat}\left(admin_{j}\right)=f_{struc}\left(admin_{j}\right)+f_{unstruc}\left(admin_{j}\right)$$

For these analyses, we assumed an uninformative prior distribution on model parameters to allow the data to drive model results. As no previous literature or data exist to inform our expectations of the spatial distribution of adolescent motherhood in Nepal at this administrative unit, there was insufficient evidence to otherwise inform a prior distribution. Finally, covariates in the model included urban/rural residence, education status and wealth quintile as specified through the DHS. We chose these covariates to reflect covariates based on similar models published elsewhere exploring adolescent motherhood in East Africa, promoting comparability across regions.^34^

Model fit was compared between models accounting for spatial effect alone (Besag) versus spatial plus random effects (BYM: Besag, York and Mollié), with models performing better with the addition of random effects. Table 1 shows model comparison and fit among all three models used in analysis, as measured by Deviance Information Criterion (DIC) and marginal log-likelihood (MLL). The DIC estimate represents model trade-offs between model complexity, goodness of fit and square error scores, while the MLL represents model evidence. Comparing these models, we find that the BYM model reduced model complexity and improved fit, and we therefore used the BYM model incorporating both spatial and random effects to predict adolescent motherhood prevalence.

**Table 1:** Model comparison and fit among all three models used in analysis, as measured by DIC and MLL

Model	DIC		MLL	
	Besag	BYM	Besag	BYM
Less than 16	276.83	273.57	−200.11	−131.61
16 to 17	357.55	356.29	−242.67	−174.05
18 to 19	371.68	368.64	−250.48	−181.26
Less than 20	407.79	406.95	−282.14	−210.33

BYC, Besag, York and Mollié; DIC, Deviance Information Criterion; MIL, marginal log-likelihood.

## Findings

Results from the initial cross-tabulations are shown in table 2. Overall, 43% of the 4419 women in the sample aged 20–29 years old had given birth before they were 20 years old, with 4.8% giving birth before their 16th birthday. The percentage of women giving birth increased as the age groups increased, with around a half of all first births before the age of 20 years occurring to women aged 18–19 years.

**Table 2:** Percentage of women aged 20–29 years experiencing a first birth before 20 years disaggregated by age group, urban/rural residence, level of education, wealth quintile, marital status and geographic region

% first births by age	< 16 years	16–17 years	18–19 years	<20 years
Total first births	4.8	16.4	22.1	43.3
Urban/rural residence				
Urban	4.4	13.6	16.6	34.6
Rural	4.9	17.7	24.5	47.1
Ratio richest/poorest	*1.1*	*1.3*	*1.5*	*1.4*
Level of education				
No education	10.1	25.5	28.0	63.6
Primary	6.4	20.5	24.0	50.9
Secondary	1.9	11.9	22.4	35.2
Higher	0	1.1	3.9	5.0
Wealth quintile				
Lowest	5.8	24.5	28.6	58.4
Second	5.8	19.2	27.2	52.2
Middle	4.8	17.2	25.4	47.4
Fourth	5.4	15.5	19.5	40.4
Highest	2.9	9.2	14.0	26.1
Ratio richest/poorest	*2.0*	*2.7*	*2.0*	*2.2*
Married at time of birth	92.4	98.3	98.7	97.9
Region				
Mountain	3.4	17.7	27.0	48.1
Hill	4.5	16.0	20.1	40.6
Terai	5.5	16.5	22.3	41.6

A higher proportion of women gave birth before the age of 20 years in rural areas compared with urban (47% compared with 34%), yet this differential is less marked for the youngest age group (4.9% compared with 4.4%). However, when we examine level of education, there is a clear gradient for all age groups: 64% of women with no education gave birth before aged 20 years, compared with 35% with secondary education and 5% with higher education. The difference is particularly large for first births under 16 years: 10% of women with no education gave birth before this age compared with less than 2% of those with secondary. There is also a wealth gradient for all the age groups, with a poorest:richest quintile ratio of 2.2 for pregnancy before the age of 20 years. The vast majority (98%) reported that they were married at the time of their first birth. However, it is noticeable that this figure is markedly lower, at 92%, for the youngest (<16 years) age group.

**Figure 1:**
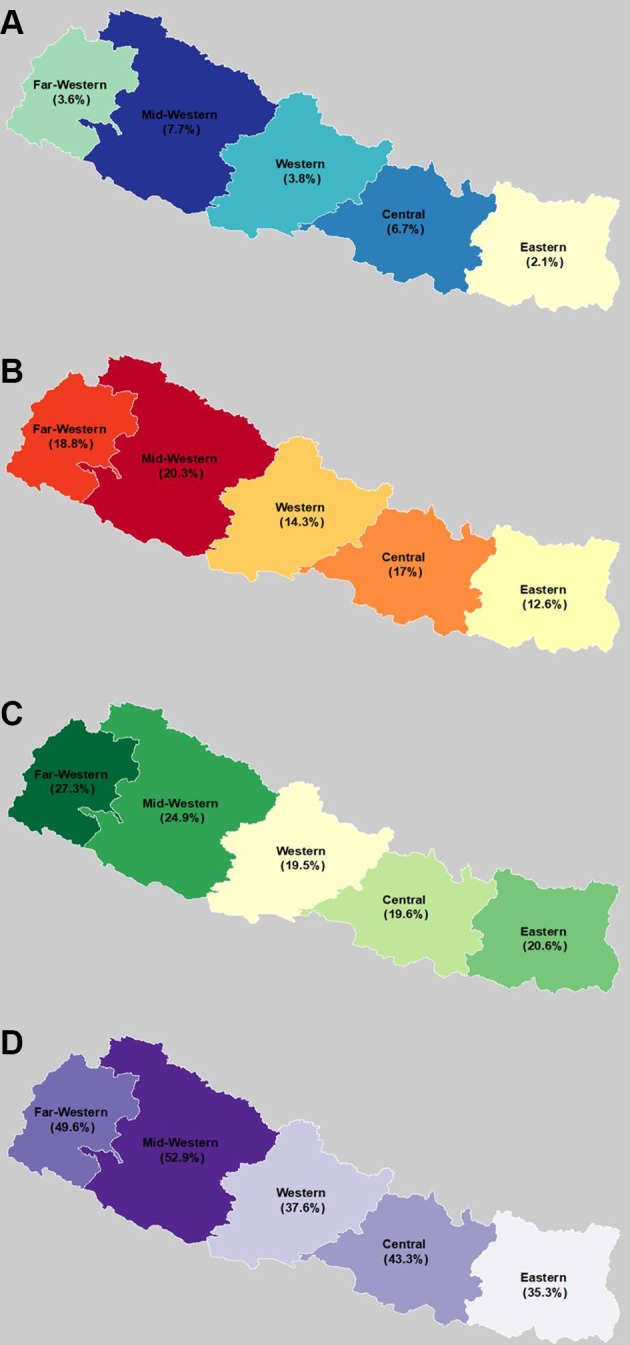
Choropleth maps by Development region for first births (A) < 16 years, (B) 16/17 years, (C) 18/19 years and (D) <20 years based women aged 20-29 years at time of survey: Nepal DHS 2011.

There are clear differences in the percentage of first births before 20 years by topographical region, with higher rates in the Mountain region. However, it is worth noting that the proportion of first births to women age<16 is higher in the Terai region. Figure 1 is a choropleth map showing the percentage of first births in each age group by development region. The overall percentage of first births before the age of 20 years is highest in the Mid and Far Western regions, followed by the Central region. However, the different age groups show slightly different patterns; for the<16 group, the highest percentages are in the Mid-Western and Central regions, with much lower percentages in the Far Western region. For the 18–19 year age group, the highest percentages are in the Far Western region. The differences are much more marked in the lower age groups than in the older age groups.

**Figure 2:**
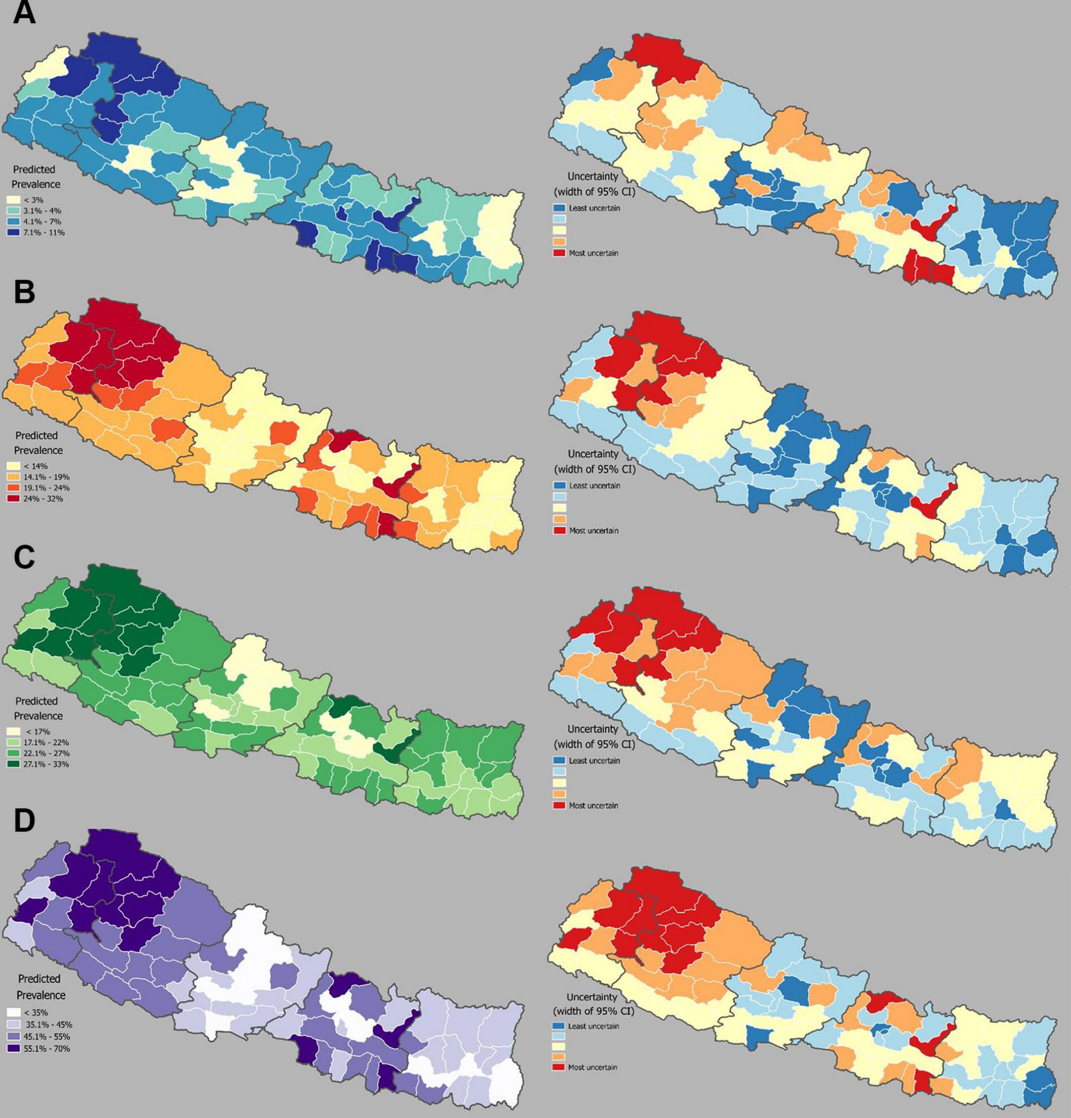
Predicted prevalence and corresponding uncertainty (width of 95% confidence intervals) of age at first birth among women (aged 20 to 29), at (A) less than 16 years old, (B) 16 to 17 years old, (C) 18 to 19 years old, and (D) less than 20 years old, Nepal DHS 2011.

When we examine the predicted prevalence maps by administrative region III (district level), we see heterogeneity for all age groups between the districts. Estimates range from 26% to 69% (see figure 2 and online supplementary material 1 for a full table of estimates by district). The predicted prevalence for first births under the age of 16 years range from 2% to 11%. Generally, the relative geographic disparities are more marked in younger age groups compared with the older^18 19^ age group: the predicted prevalence for the 18–19 age group ranges from 15% to 33%.

10.1136/bmjgh-2018-000763.supp1 Supplementary data

Bayesian methods generate a range of ‘probable’ estimates for a given outcome or ‘posterior estimate’, which varies from the point estimate of more standard frequentist approaches. Here, our primary outcomes are the mean of posterior distribution at each district, but we also report distribution statistics such as SD and width of the 95% credible interval, providing insight into the possible ranges that ‘true’ estimates might fall within. For example, since our estimates are prevalence estimates which fall between 0 and 1, an estimate with 100% uncertainty would have an associated wide 95% credible interval, where the ‘true’ estimate might fall anywhere between 0 and 1. Therefore, each of the 75 districts in our study area have a posterior range comprising of a mean, SD and credible intervals, which are reported in table 3.

**Table 3:** Distribution statistics of the SD estimates for prevalence of adolescent first births for the 75 districts for each age group

Age group	Minimum	First quartile	Median	Mean	Third quartile	Maximum
<16 years	0.0058	0.0089	0.0116	0.0130	0.0167	0.0263
16–17 years	0.0181	0.0136	0.0161	0.0172	0.0201	0.0309
18–19 years	0.0142	0.0205	0.0210	0.0212	0.0238	0.0309
<20 years	0.02680	0.04108	0.04700	0.04708	0.05453	0.06416

High prevalence districts for first births< 20 years tend to be clustered within the Mid-West and Far-West Regions, with a lesser extent in the Central Region. However, there are also distinct differences with these regions. For instance, if we examine the map for first births<20 years in figure 2, we see that there are districts in the Central region that are in both the highest and lowest prevalence categories. For the <16 and 16–17 group, we see a number of clearly defined high prevalence districts in the Mid/Far Western and Central regions. It is hard to see much clear association with the differentials at topographic region (eg, Mountain, Terai and Hill) for any of these age groups. The high prevalence of early first births in the Western regions cuts across all these terrains, but is clearer in the central region of the < 16 age group, where these specific births are concentrated in the Terai districts of this region.

## Discussion

Our findings suggest that nearly 40% of women in Nepal aged 20–29 years have given birth before the age of 20. Around half of these are aged 18–19 years at the time of their first birth, but around 5% of our sample have given birth before the age of 16 years. Our initial cross tabulations confirm that adolescent pregnancy is most common among the poorest, least-educated and rural dwellers. However, unlike other studies in sub-Saharan Africa,^32 37^ we did not find that the inequalities are strikingly greater for the youngest age group, with the exception of education.

Our geographical analysis showed marked differences in the percentages of first births for all three adolescent age groups within both topographic and development regions. Comparison between our district-level prevalence maps reflects the patterns seen for development regions, and to a much lesser extent, topographic regions. However, geospatial modelling identifies differences within these regions at a higher spatial resolution and more policy relevant scale, such as the district level, and provides a much more nuanced picture.

Adolescent pregnancy is clearly associated with poverty, lack of education and rural residence. However, model fit improved when comparing spatial and non-spatial models, suggesting additional underlying geographic drivers. For instance, our findings suggest a high proportion of adolescent births within the central Terai, which is likely to be underpinned by very high levels of early marriage.^38^ While poverty is a determinant of early marriage, it is also underpinned by a number of other social and cultural factors, including dowry practices, which are not normally captured in demographic surveys.^39^ Brown^40^ also describes how topographic and access factors affect gender roles in communities, and these factors could also influence early marriage and subsequent pregnancy. Such factors are not captured simply using ‘urban/rural’ distinctions in primarily rural countries such as Nepal, as this does not give an adequate measure of remoteness. In addition, these data cover the period of the Nepalese Civil War (1996–2006).^41^ Conflict intensity was greatest in the Mid-Western and Far Western region, which demonstrate high levels of adolescent first births. Conflict is often associated with increased early marriage in cultures where it is already the norm^42^ and Williams *et al* demonstrated a clear link between conflict intensity and reduction in age of marriage in Nepal.^43^

It is important to note that in some areas, a significant proportion of women have given birth before the age of 16 years. This is associated with high risks to the health of both mother and infant.^18 19 44^ The geospatial model provides policymakers with estimates of where rates of early motherhood occur at a spatial resolution not previously reported. It is able to point to some districts where very early first births seem to be common (up to 11%), suggesting the need for further investigation to confirm this and understand the drivers of these patterns. Our analyses enable efforts and resources to be targeted where they are most needed. In addition, it was noted that nearly 10% of this group reported they were unmarried at the time of first birth, greater than for older adolescents. In contexts of universal marriage, these women are likely to be exposed to stigma and exclusion, making it particularly important that the context of these births is investigated further.

Reducing adolescent births in Nepal is likely to require a holistic, multifaceted response, which both addresses the underlying determinants and improves access to appropriate sexual health information and services. Addressing early marriage will be a key factor, but it is worth noting that reductions in early marriage do not necessarily translate into similar gains in adolescent pregnancies. There is usually a long gap before consummation for marriages taking place at a very early age. However, as the age has risen, the time between marriage and consummation has actually reduced, resulting in less impact on age at first birth.^12^

Improving access to sexual health information and services is a priority. The National Adolescent Sexual and Reproductive Health Programme in Nepal has launched counselling and family planning provision in 63 of Nepal’s 73 districts, and comprehensive sexuality education has been introduced in schools.^45^ However, there are still major barriers to access to contraception for young people, including cultural issues and gender power imbalance, geographical remoteness, lack of knowledge and financial constraints.^45^ As age of marriage has risen, there is also an increasing window for young people to engage in premarital sex:^46^ this group is likely to be highly stigmatised in a conservative culture such as Nepal and will require confidential services tailored to their needs if poor reproductive health outcomes are to be avoided.

Our study has a number of limitations. First, as discussed in our findings section there are uncertainly levels associated with our district level estimates (see table 3), which tended to be relatively higher in the western regions, probably due to smaller sample sizes. It is also important to note that these are modelled estimates using statistical inference with data designed to be representative at a coarser geographical scale. As with all analysis of this type, results should therefore be interpreted with caution without further model validation efforts. However, DHS endorses the use of its data for this type of geostatistical modelling and are currently using similar approaches themselves to produce modelled surfaces.^13^

The data itself has a number of limitations. First, it should be recognised that data on socioeconomic status and geographical location are at the time of survey collection, not at the time of birth. As there may be some years between the birth and data collection, socioeconomic circumstances could have changed or women could have moved district between the time they gave birth and the time of survey. In addition, the data relies on respondents’ recall for age at first birth. There is evidence that social-desirability bias and concern around potential censure may deter women from reporting very early ages at birth, but this appears to be mostly when respondents are under 20 years old, rather than in the 20–29 year age group used in this study.^47^ Finally, the data are now somewhat dated. A new DHS survey was carried out in Nepal in 2016, but the data had not yet been released at the time this paper was under development. Nonetheless, it is important that further analysis be carried out to ensure a contemporaneous understanding of the situation.

## Conclusion

Our study demonstrates important inequities in adolescent first births both by socioeconomic status and by geography. In some areas, there are still alarmingly high levels of adolescent first births, including births to very young adolescents under 16 years. The use of Bayesian geospatial modelling techniques offers a more nuanced picture of differentials in adolescent first births and inequities between districts. Such geospatial techniques have important uses in supporting policymakers and planners in developing programmes to address adolescent fertility and enable resources and efforts to be directed where they are most needed.
