# Supplementary material for: Using geospatial modelling to estimate the prevalence of adolescent first births in Nepal
Source: BMJ Glob Health. 2019 Jul 1;4(Suppl 5):e000763. doi: 10.1136/bmjgh-2018-000763 (PMC6606082; doi:10.1136/bmjgh-2018-000763)
Supplement: Supplementary data [file bmjgh-2018-000763supp001.pdf]

Supplementary Material S1: Predicted prevalence by administrative III units of first births for women aged 20-29years (a) <16 years, (b) 16/17 years, (c) 18/19 years and (d) <20 years: Nepal DHS 2011

		Predicted prevalence estimates			
Region	District	<16 years	16/17 years	18/19 years	<20 years
Central	Nuwakot	0.055	0.112	0.145	0.302
Central	KavrePalanchok	0.055	0.112	0.145	0.301
Central	Lalitpur	0.070	0.131	0.152	0.350
Central	Kathmandu	0.051	0.121	0.165	0.274
Central	Bhaktapur	0.032	0.115	0.178	0.281
Central	Chitwan	0.057	0.134	0.186	0.405
Central	Dolakha	0.038	0.136	0.193	0.353
Central	Makwanpur	0.063	0.171	0.205	0.464
Central	Sindhuli	0.063	0.185	0.214	0.456
Central	Parsa	0.093	0.211	0.215	0.566
Central	Rautahat	0.035	0.152	0.230	0.431
Central	Bara	0.039	0.162	0.237	0.455
Central	Mahottari	0.099	0.231	0.243	0.534
Central	Sindhupalchok	0.038	0.182	0.247	0.491
Central	Sarlahi	0.060	0.208	0.252	0.509
Central	Dhanusa	0.111	0.259	0.254	0.665
Central	Dhading	0.035	0.192	0.263	0.517
Central	Rasuwa	0.067	0.259	0.282	0.552
Central	Ramechhap	0.084	0.283	0.295	0.624
East	Dhankuta	0.044	0.133	0.184	0.346
East	Morang	0.034	0.122	0.184	0.364
East	Sunsari	0.040	0.134	0.185	0.381
East	Udayapur	0.041	0.141	0.194	0.346
East	Jhapa	0.038	0.141	0.194	0.304
East	Ilam	0.019	0.118	0.206	0.261
East	Bhojpur	0.033	0.144	0.209	0.380
East	Siraha	0.090	0.205	0.209	0.508
East	Khotang	0.025	0.134	0.227	0.375
East	Taplejung	0.022	0.133	0.227	0.381
East	Terhathum	0.022	0.136	0.229	0.362
East	Panchthar	0.020	0.133	0.231	0.383
East	Sankhuwasabha	0.032	0.150	0.233	0.420
East	Saptari	0.056	0.189	0.237	0.456
East	Solukhumbu	0.031	0.177	0.255	0.438
East	Okhaldhunga	0.042	0.206	0.266	0.476
Far-Western	Baitadi	0.039	0.141	0.210	0.404
Far-Western	Kanchanpur	0.053	0.158	0.214	0.437
Far-Western	Kailali	0.052	0.157	0.214	0.452

Far-Western	Darchula	0.028	0.159	0.262	0.459
Far-Western	Doti	0.054	0.217	0.273	0.526
Far-Western	Dadeldhura	0.056	0.229	0.279	0.576
Far-Western	Bajura	0.057	0.243	0.286	0.589
Far-Western	Achham	0.064	0.272	0.308	0.605
Far-Western	Bajhang	0.080	0.304	0.328	0.665
Mid-Western	Dang Deokhuri	0.070	0.168	0.213	0.486
Mid-Western	Banke	0.055	0.171	0.222	0.487
Mid-Western	Bardiya	0.060	0.186	0.237	0.467
Mid-Western	Surkhet	0.041	0.154	0.238	0.513
Mid-Western	Pyuthan	0.031	0.158	0.239	0.391
Mid-Western	Rukum	0.031	0.159	0.249	0.496
Mid-Western	Dailekh	0.071	0.228	0.257	0.541
Mid-Western	Salyan	0.029	0.164	0.258	0.469
Mid-Western	Dolpa	0.041	0.185	0.263	0.516
Mid-Western	Rolpa	0.049	0.201	0.265	0.544
Mid-Western	Jumla	0.059	0.242	0.286	0.604
Mid-Western	Jajarkot	0.056	0.238	0.286	0.600
Mid-Western	Mugu	0.077	0.285	0.307	0.621
Mid-Western	Kalikot	0.090	0.296	0.313	0.685
Mid-Western	Humla	0.088	0.309	0.320	0.623
West	Manang	0.057	0.114	0.149	0.314
West	Gulmi	0.057	0.114	0.149	0.314
West	Mustang	0.057	0.114	0.150	0.317
West	Kaski	0.055	0.125	0.155	0.317
West	Rupandehi	0.049	0.124	0.171	0.325
West	Gorkha	0.047	0.136	0.189	0.375
West	Syangja	0.028	0.121	0.196	0.338
West	Baglung	0.031	0.137	0.206	0.371
West	Parbat	0.022	0.114	0.210	0.312
West	Tanahu	0.035	0.146	0.219	0.414
West	Palpa	0.026	0.138	0.226	0.408
West	Nawalparasi	0.031	0.144	0.232	0.427
West	Kapilvastu	0.036	0.153	0.236	0.408
West	Arghakhanchi	0.028	0.151	0.241	0.433
West	Myagdi	0.028	0.160	0.252	0.464
West	Lamjung	0.042	0.199	0.267	0.485
